# A common short-term memory retrieval rate may describe many cognitive procedures

**DOI:** 10.3389/fnhum.2014.00126

**Published:** 2014-03-07

**Authors:** Evie Vergauwe, Nelson Cowan

**Affiliations:** Department of Psychological Sciences, University of MissouriColumbia, MO, USA

**Keywords:** short-term memory, working memory, attention, retrieval, refreshing, memory search

## Abstract

We examine the relationship between response speed and the number of items in short-term memory (STM) in four different paradigms and find evidence for a similar high-speed processing rate of about 25–30 items per second (∼35–40 ms/item). We propose that the similarity of the processing rates across paradigms reflects the operation of a very basic covert memory process, high-speed retrieval, that is involved in both the search for information in STM and the reactivation or refreshing of information that keeps it in STM. We link this process to a specific pattern of rhythmic, repetitive neural activity in the brain (gamma oscillations). This proposal generates ideas for research and calls for an integrative approach that combines neuroscientific measures with behavioral cognitive techniques.

An important feature of human information processing is short-term memory (STM), the ability to retain a small amount of information in a highly accessible state for a short time. The capacity of STM is limited to a certain number of items, and a key issue in cognitive psychology is the reason why STM is limited. Here we suggest that, over the last 40–50 years, at least four different paradigms have been developed that provide insights into the temporal properties of STM. Despite the wide variety of paradigms, we observed an intriguing similarity in a high-speed processing rate of about 25–30 items per second, which can be inferred from the relationship between response speed and memory load. We propose that the similarity of the processing rates across paradigms may reflect a basic covert memory process (i.e., a memory process that is inferred from the pattern of recall performance across certain conditions, rather than being directly observable), high-speed retrieval, which can be used for either recognition of a probe item or reactivation (refreshing) of an item for the sake of maintenance. We also link this process to recent developments in the neuroscientific literature and discuss implications for future research.

## The relationship between response speed and memory load in four paradigms

**Figure 1:**
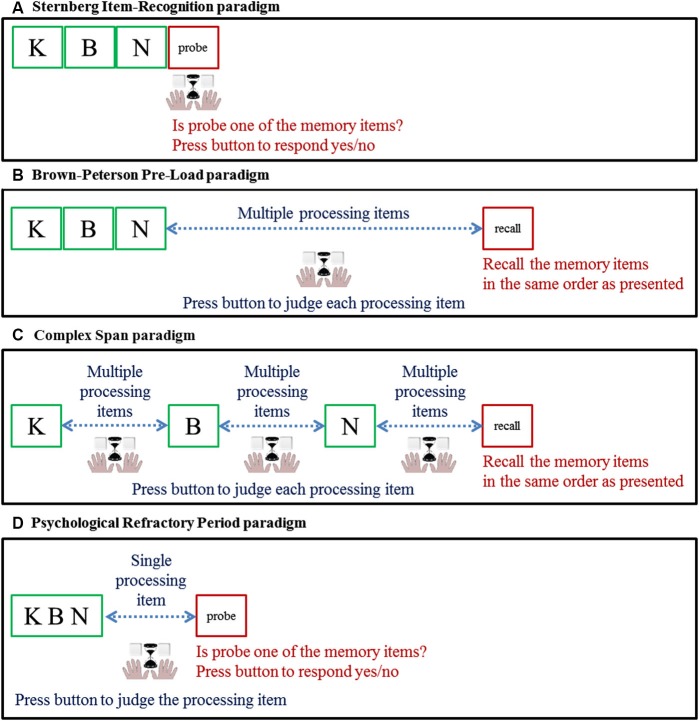
**Schematic presentation of four paradigms providing insights in the relation between response speed and memory load.** In the example, participants are presented with three letters to be maintained: K, B and N. The hand symbol together with the hourglass refers to a response given by pressing a button for which the speed is the variable of interest here. In **(A)** the Sternberg Item-Recognition paradigm, we examined speed of response to probe as a function of the number of memory items; in (B) through (D) we examined speed of response to processing items as a function of the concurrent number of items in memory; (**B)** Brown-Peterson Pre-Load paradigm; **(C)** Complex Span paradigm; **(D)** Psychological Refractory Period paradigm.

**Figure 2:**
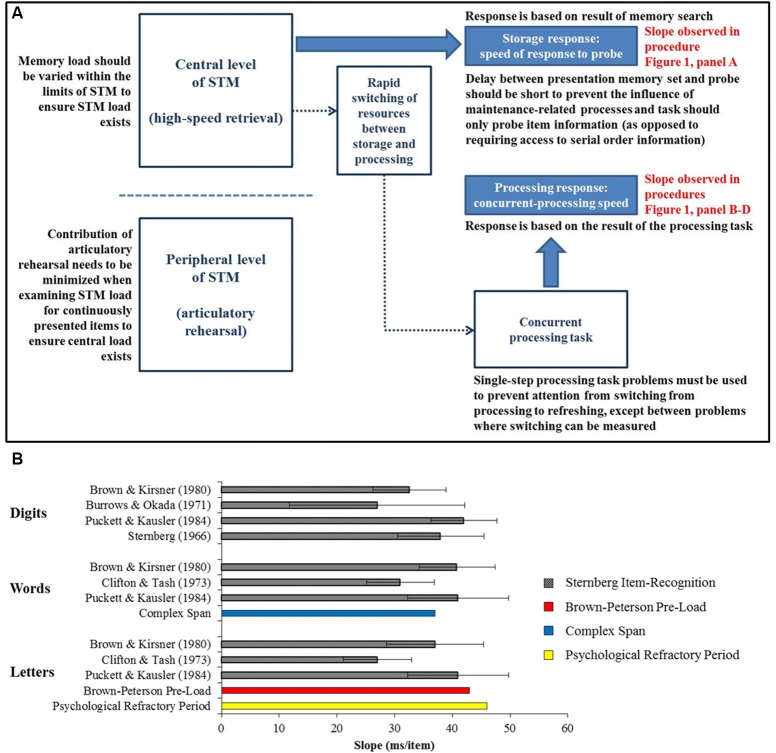
**(A)** Schematic presentation of cognitive interpretation of the observed processing rate together with the boundary conditions (in black) that must be met to observe a clear relationship between memory load and response latency. Two different levels in STM are presented: a central level and a peripheral level. For verbal stimuli, the peripheral level offers an alternative maintenance mechanism (articulatory rehearsal), the use of which should be minimized when examining STM load for continuously presented items. **(B)** Estimates of STM retrieval slope for three kinds of verbal materials based on *(1)* the Sternberg Item-Recognition paradigm (gray bars), *(2)* the Brown-Peterson Pre-Load paradigm (red bar, second from the bottom), *(3)* the Complex Span paradigm (blue bar, last in the Words cluster), and *(4)* the Psychological Refractory Period (yellow bar, bottom). For the Sternberg Item-Recognition paradigm, the figure only includes studies that provided the information necessary to calculate 95% confidence intervals (represented by error bars). For the other paradigms, the unweighted average across studies mentioned in the text is presented.

After the seminal article of Miller (1956) on STM capacity limitations, human STM research mainly investigated the determinants of failure of STM by focusing on accuracy and error patterns in simple memory tasks. In the late 1960s, however, a complementary approach became increasingly popular. This approach consisted of studying how much time participants need in order to succeed in simple memory tasks. Specifically, Saul Sternberg studied how much time participants needed in order to indicate whether a probe item was present in a small set of memorized elements (Sternberg, 1966, 1969a). The rationale was that, if the information in memory is needed to select the appropriate response, then the time taken to give that response will reveal something about the process by which one is searching in memory for that information. In order to explore the timing of memory search, Sternberg proposed what we term the Sternberg Item-Recognition paradigm (Figure 1A). Although it is still the standard paradigm to investigate memory search rates, at least three other paradigms can be identified as providing insights into the temporal properties of STM (Figures 1B–D); all show a positive relation between the number of items to be retained in STM (memory load) and the time it takes to respond to a probe item (response latency). Figure 2A provides an overview of what, based on our review, seem to be necessary boundary conditions that must be met to observe a clear positive relation between memory load and response latency. In what follows, only studies that met these conditions are reported and, when interpreting the observed common processing rate, we will explicitly address the role of these boundary conditions.

For the sake of comparison, in the following review, we only included data of experiments that used simple verbal stimuli to be memorized (digits, letters and words), using healthy young adults as participants. We only included studies that provide the information necessary to examine a particular key index of the rate of retrieving information from STM, the slope of the function that relates response latency to STM load. Consequently, we only considered studies that included at least two different levels of memory load and that either reported the slope of the relation of interest or reported response latency for each memory load condition so that we could estimate the slope (averaged across positive and negative responses). Despite the fact that the four paradigms differ quite substantially in their methodology (see Figure 1), we identified a similar processing rate across them.

## The Sternberg item-recognition paradigm

The variable of interest is the speed with which participants decide whether the probe is a member of set of items held in STM by pressing, as quickly as possible without making errors (Figure 1A). It is assumed that this decision requires people to scan through the content of STM to compare the probe with each item in memory. Delay of the response is interpreted as the operation of this time-consuming covert memory search. The classical finding is that response times increase linearly with the size of the memory set with a slope of about 35–40 ms per additional item in memory (Sternberg, 1966, 1969a). The slope of this function is assumed to reflect the time it takes to retrieve a single item from STM. These classic findings of Sternberg launched a very productive line of investigation on memory search in cognitive science, with an overwhelming number of studies testing the original paradigm and variants of it. Because of the limited space here, the included studies using this paradigm were limited to the visual modality for presentation of both memory items and test items. On average, response latency increased at a rate of 37 ms per additional item held in memory.^1^

## The Brown-Peterson pre-load paradigm

In the paradigm developed by Brown (1958) and Peterson and Peterson (1959), a few stimuli to be remembered are followed by a processing task that is different enough to avoid material-specific interference, but challenging enough to prevent attention to the memoranda or rehearsal of them (Figure 1B). The main finding was that memory is lost rapidly across about 30 s. The variable of interest here, though, is processing speed on the concurrent processing task that precedes recall. Slowing down has been shown in several studies comparing response speed under concurrent memory load with response speed without a concurrent load (e.g., Shulman and Greenberg, 1971; Baddeley and Hitch, 1974; Logan, 1978). It is assumed that, during the retention interval filled with processing, people engage in storage-related activities. When processing and storage both rely on attentional resources, storage-related activities are expected to postpone concurrent processing activities. Methodological details can be found in Footnote 2.^2^ Vergauwe et al. (2014) found that response latency increased linearly at a rate of 43 ms per additional item held in memory.

## The complex span paradigm

In this paradigm, the presentation of items to be remembered is interleaved with items to be processed (Figure 1C). The purpose was originally to assess the capability of working memory under the assumption that storage and processing share a common cognitive resource so that both of them must be engaged in order for capability to be assessed (e.g., Daneman and Carpenter, 1980). The variable of interest for the present purposes is processing speed on the concurrent processing task. Several studies have shown longer response latencies in later processing phases (high memory load), compared to the first processing phase (low memory load; e.g., Friedman and Miyake, 2004; Chen and Cowan, 2009). As for the Brown-Peterson pre-load paradigm, the underlying assumption is that slower processing reflects resource-sharing between attention-demanding processing and storage activities. Methodological details can be found in Footnote 2. Jarrold et al. (2011) found linear trends across the successive processing phases showing that response latency increases at an average rate of 37 ms per additional item held in memory (41 ms in Experiment 1 and 33 ms in Experiment 2).

Another potential variable of interest, but one that requires further work, is the time it takes to retrieve the next item to be recalled. Cowan (1992) measured the timing of spoken recall for simple digit span in children and proposed that each inter-word pause reflects a process of search through working memory to find the next digit to be recalled. Subsequent work (Cowan et al., 1998) showed that the inter-word pauses for correctly-recalled lists did increase in approximately a linear fashion with increasing list length, in children in first grade (84 ms/item), third grade (58 ms/item), and fifth grade (25 ms/item). In adults, further work is needed to establish the scanning rate. One might worry that verbal rehearsal processes would play a role, though a relation between the spoken recall rate and search rates based on the scanning paradigm was demonstrated by Cowan et al. (1998) and by Hulme et al. (1999). In complex span, presumably rehearsal processes have been interrupted by the processing task. Recall in these tasks, however, might involve more than a simple search, for example an attempt to use the processing task as a context to retrieve the list items. Thus, Cowan et al. (2003) noted that inter-word pauses in the responses lasted 4–10 times longer than in simple span.

## Psychological refractory period paradigm

This paradigm (Welford, 1952; Pashler, 1994) usually combines two processing tasks requiring two responses in succession on a single trial. The original point was to explore processing demands by studying how the processing for the first response delayed the second response. In the task variants of interest here, memory demands are combined with processing demands. After the memory set is presented, at various stimulus-onset asynchronies (SOAs), a single stimulus pertaining to the processing task is presented, to which a speeded response is required (Figure 1D). Some of these studies also manipulated the size of the memory set, which makes them of particular interest here. The finding of interest here is that the single speeded response took longer as more items were held in memory concurrently (e.g., Jolicoeur and Dell’Acqua, 1998; Stevanovski and Jolicoeur, 2007). Again, the underlying assumption is that processing and storage interfere with each other because they rely on a common attentional resource, resulting in slower processing. Methodological details can be found in Footnote 3.^3^ Processing took about 46 ms longer per additional item in memory (32 and 60 ms in Stevanovski and Jolicoeur, 2007, in Experiments 2 and 3, respectively).

## Empirical summary

We have identified a pattern that holds across four different paradigms: response speed slows down at a rate of about 30–40 ms per additional simple verbal item in memory (see Figure 2B). The similarity across the paradigms suggests strongly the existence of a high-speed processing rate in STM of about 25–30 items per second (the equivalent of 40–33 ms/item).

Previous studies have pointed out the similarity between the processing rates observed in the complex span paradigm and the Sternberg item-recognition paradigm (Jarrold et al., 2011), and between the rates observed in the Brown-Peterson pre-load paradigm and the Sternberg item-recognition paradigm (Vergauwe et al., 2014). The present contribution is to note the similarity of processing rate across a wider range of procedures, and to propose a cognitive interpretation of this high-speed processing rate, in the next section.

## Cognitive interpretation of high-speed processing rate in human short-term memory (STM)

We interpret the identified processing rate as reflecting the operation of a very basic covert memory process, retrieval from STM. In this view, although information retrieval and maintenance are typically referred to as different stages in STM, they are proposed to rely on the same process. When responding to a probe in the Sternberg task, high-speed retrieval is used in the service of memory search. It brings items in the focus of attention so that one can check whether it matches the probe. The slope observed in this task reflects directly the use of high-speed retrieval. In the three remaining paradigms, high-speed retrieval is used in the service of memory maintenance; it brings items in the focus of attention so that the information gets reactivated or refreshed. When high-speed retrieval and concurrent processing share a common resource (attention), the use of high-speed retrieval influences concurrent processing speed so that response latency increases for each additional item that is maintained. Under the assumption that maintenance is accomplished through sequential reactivation of information in a cumulative fashion, starting from the first list item and proceeding in forward order until the end, the observed rate reflects the rate at which items are reactivated in STM. In the Sternberg task, it is assumed that the presentation of the probe initiates a complete cycle through STM. In the other paradigms described here, storage is combined with a self-paced processing task and the idea is that a complete cycle of refreshing is interpolated before attention-demanding processing takes place. Thus, provided that participants aim at performing well on the memory task, attention is first used for a complete cycle through STM before it is shifted to the next processing stimulus. It is possible, though, that the same assumption might not hold in tasks in which the processing task is to be performed at a predefined pace (i.e., computer-paced). In these tasks, every processing item is typically followed by a variable period of free time during which refreshing can take place in a continuous matter. If the process of refreshing is exhaustive in nature, one might expect that, upon the presentation of the next processing item, on average only half of the items in STM would still need to be reactivated. Slopes relating response times to memory load would then reflect the amount of time it takes to scan half of the number of items in STM.

A schematic presentation of our cognitive interpretation of the observed processing rate is shown in Figure 2A. Two different levels in STM are presented: *(1)* a central level that is domain-general in nature, closely related to attention, and *(2)* a peripheral level that is domain-specific in nature and independent from the central level. High-speed retrieval is used at the central level to bring information into the focus of attention.

Together with the observation of Cowan et al. (1998) that retrieval rate as measured in a search task correlates with memory span, the identification of a rapid retrieval rate across several paradigms is directly relevant to the long-standing debate regarding the nature of the severe capacity limit of STM. Theoretically, the capacity limit of STM might reflect the number of items that can be active simultaneously within a given time-window. If one assumes that there is a limited time-window within which the items need to be reactivated so that all of them can be retained, then the capacity limit of STM would depend on the retrieval rate with faster rates resulting in more items reactivated within the fixed time-window. A similar idea was proposed by Cavanagh (1972) who showed an inverse relation between STM span and memory search rate for different materials. The speed of retrieval in STM also indicates that STM functions in a way that is much more rapid and dynamic than most people would think. Importantly, we consider this rapid retrieval rate to be independent of the slower verbal rehearsal rate that relies on covert speech, even though both might serve the same goal of maintaining information in STM (see Cowan et al., 1998; Hulme et al., 1999; Camos et al., 2011).

Note that although the idea of a limited time-window implies the existence of time-based forgetting in STM, it is not incompatible with interference-based forgetting. When items are not reactivated in time, forgetting might occur either because memory traces have decayed or because newer representations have overwritten previous ones or have become confusable with the previous ones. The degree of confusability might then depend on the number of features that are shared between the representations in STM. Moreover, Ricker and Cowan (2014) have recently shown that the process of consolidation influences the observed rate of forgetting over time with more consolidation leading to slower rates of time-based forgetting. This finding indicates that the relationship between STM capacity, retrieval rate and decay rate might depend on the robustness of the trace. Also, the length of the critical time-window might differ between individuals and this possibility needs to be taken into consideration when focusing on the relation between high-speed retrieval and STM capacity across individuals.

## Boundary conditions

There are studies in which the slope of the relationship between response speed and memory load was substantially smaller than the proposed constant of about 37 ms per item in normal adults. For example, in a Sternberg task, Banks and Atkinson (1974) forced participants to respond so quickly that they made a lot of errors. A flatter slope may occur when speed is stressed at the expense of accuracy because participants base their response on a feeling of familiarity, which can occur for all items in parallel, rather than on a more time-consuming but accurate item-by-item memory search. Burrows and Okada (1975) showed that the Sternberg slope changes at the limits of STM with a shallow slope of 13 ms when considering memory loads ranging between 8 and 20 words, for which the only viable mechanism might be familiarity. In the processing times within complex span, but using viewing or reading times rather than simple reaction times (RT) slopes across memory loads vary considerably (e.g., Engle et al., 1992; Friedman and Miyake, 2004). Viewing or reading might be covertly interrupted for refreshing, evading measurement. There are also studies in which the slope of the relationship between response speed and memory load was larger than the proposed constant of about 37 ms per item. RT slopes across memory loads are considerably steeper (up to about 100 ms per item) in studies that use Sternberg-like tasks in which participants need to have access to serial order information in order to judge the probe correctly, as opposed to the typical Sternberg task in which access to item information is sufficient (e.g., Sternberg, 1969b; Ravizza et al., 2011; Majerus et al., 2012). Furthermore, studies in which a delay of several seconds was inserted between the presentation of the memory set and the presentation of the probe also reported somewhat steeper slopes (about 50–55 ms per item; e.g., Cairo et al., 2004; Chen and Desmond, 2005). Maintenance-related processes such as verbal rehearsal might take place during this delay and as such, influence the observed retrieval rate at the end of the trial. We suggest boundary conditions to observe a clear, positive relation between memory load and response latency, as presented in Figure 2A.

## Relating high-speed retrieval in short-term memory (STM) to oscillations in the brain

Recent neuroscientific developments lead to a view of retrieval rate as governed by oscillations (rhythmic, repetitive neural activity; e.g., Lisman and Jensen, 2013). In the dual oscillation model of STM (Lisman and Idiart, 1995) it is proposed that the features of one item are active at the same time and are represented by a group of neurons that fire in the same gamma cycle (30–80 Hz). Next, the features of a second item are active at the same time and represented by the second gamma cycle within the same theta cycle (4–8 Hz). Lisman and Idiart (1995) linked the Sternberg slope to the duration of a gamma cycle. One item would be searched each time its gamma cycle of neural activity occurred. They also suggested that STM capacity limits could be determined by the number of gamma cycles that fit into one theta cycle. Given current uncertainties in these figures, this neural theory is reasonably compatible with a cognitive proposal by which STM capacity depends on the number of items that can be reactivated within a given time-window so that several items can be retained in a refreshed state simultaneously. Each gamma cycle would allow the refreshment of one item in STM. Our empirical retrieval rate of 37 ms/item would correspond to a gamma cycle of 27 Hz and would allow 3–6 items per theta cycle.

Thus, we propose to extend the view of Lisman and Idiart so that it encompasses our expanded function of high-speed retrieval. In this view, refreshing consists in the rapid reactivation of a limited number of items at a rate that reflects the length of one gamma cycle per item. In support of a link between STM maintenance and gamma oscillations, changes of oscillatory activity in the human gamma frequency band related to STM retention have been observed (e.g., Tallon-Baudry et al., 1998; Jokisch and Jensen, 2007; Meltzer et al., 2008) and Howard et al. (2003) showed that, in a Sternberg-type task, gamma power during retention was higher for larger memory sets. Furthermore, Roux et al. (2012) showed a relation between gamma-band activity and memory load in a left prefrontal area of the brain that has been associated with refreshing (e.g., Johnson et al., 2005). In this study, a number of red disks were displayed in different locations. After a short delay, a single red disk was shown and participants decided whether its location matched one of the study locations. An increase in gamma-band power between load 3 and load 6 was observed during the delay and this increase correlated with memory performance. Finally, Kamiński et al. (2011) found a negative correlation between individual’s STM performance and gamma cycle length. This is exactly the kind of relationship one would expect if STM capacity depends on the number of items that can be reactivated within a given time-window with each gamma cycle allowing the reactivation of one item in STM.

## Conclusion and outlook

The current proposal is novel in at least two ways. First, it proposes that the identified high-speed processing rate of about 27 items per second across four different procedures might reflect the operation of a very basic process of high-speed retrieval that serves both memory search and attention-based refreshing in STM. Thus, the attentional component of memory search and refreshing is proposed to be the same. This does not preclude the theoretical possibility that refreshing is equivalent to retrieval plus some additional operations; it only restricts these additional operations to a set of operations that do not require attention. Second, it proposes that this general process might be associated with gamma brain oscillations. We believe that our proposal has the potential of providing novel insights into the significant questions of how information is maintained in STM and why it’s capacity-limited. The proposal is based on a limited number of studies at this point and further research is needed, but the present proposal suggests several clear directions for further research.

Behavioral research should aim at testing the unique predictions that follow from our proposal. First, memory search and maintenance are proposed to rely on the same STM retrieval process. One direction is to look for interference patterns between both processes. The results of ongoing research of ours suggest that the memory search slope varies as a function of the time available to refresh memoranda. Another test of our proposal would be to compare the processing rates across the four paradigms in a within-participants design. This might also help us understand whether variations in the processing rate between procedures and materials are meaningful. Second, refreshing of a series of items is proposed to be enacted by consecutive gamma oscillations. Does the order of spontaneous refreshing follow the order of presentation? Can the distance between individual items in STM be described in terms of the number of gamma cycles that separate them? When a set of multiple items is successfully chunked into a few chunks, can we observe a decrease in the number of gamma cycles one needs to run through in order to refresh the entire set? In addition, future research should aim at testing the universality of the identified retrieval process by searching whether a similar processing rate can be observed in other paradigms and by examining response time distribution data. Another remaining question is whether transferring new external information into STM would occur at the same rate. The results of some studies suggest a slower rate of consolidation of about 200–250 ms per item in the Brown-Peterson pre-load paradigm (e.g., Jarrold et al., 2011; Vergauwe et al., 2014). This rate matches the length of theta cycles which have been linked to encoding new information (e.g., Klimesch, 1999). Finally, neurophysiological and cognitive approaches should be integrated to examine whether the length of gamma cycles and retrieval rate are influenced by the same factors (experimental, individual, developmental, clinical), and whether externally induced changes in gamma frequency (e.g., through magnetic stimulation of the brain) affect STM speed and capacity.

## Conflict of interest statement

The authors declare that the research was conducted in the absence of any commercial or financial relationships that could be construed as a potential conflict of interest.

## Footnotes

^1^The average of 37 ms/item refers to an unweighted average across 8 slopes for digits (*M* = 36 ms/item), 5 slopes for words (*M* = 36 ms/item) and 13 slopes for letters (*M* = 38 ms/item). When only taking into account the studies that provide information to calculate 95% confidence intervals (i.e., the studies included in the lower panel of Figure 2), the unweighted average is 36 ms/item.

^2^In the Brown-Peterson pre-load paradigm and the complex span paradigm, a difference is typically made between the RT for the individual’s first response in the processing phase, referred to as first processing times and the mean of all subsequent RTs in that processing phase, referred to as subsequent processing times. While longer first processing times have been attributed to the consolidation of memory traces, longer subsequent processing times are typically attributed to the maintenance of memory traces (e.g., Engle et al., 1992; Jarrold et al., 2011). Because our focus is on the maintenance process rather than on consolidation, the current manuscript only reports analyses that concern subsequent processing times. The slopes reported for the complex span paradigm concern an average across different list lengths (from 4 to 7 words).

^3^We included only conditions in which participants are required to recall all of the information after this speeded response because, only in those conditions, participants are required to make the processing response while keeping the information active in memory. Furthermore, we only included studies in which the effect of memory load on response time did not depend on SOA because only in those studies, the effect can be interpreted as a cost related to maintaining information in STM rather than consolidating information into STM.
